# DiaGuide-LLM—Using large language models for patient-specific education and health guidance in diabetes

**DOI:** 10.3389/frai.2025.1652556

**Published:** 2025-11-28

**Authors:** Kristin Skjervold, Henriette Nordahl Sævig, Helge Ræder, Arvid Lundervold, Alexander Selvikvåg Lundervold

**Affiliations:** 1 Department of Biomedicine, University of Bergen, Bergen, Norway; 2 Department of Clinical Science, University of Bergen, Bergen, Norway; 3 Department of Pediatrics, Haukeland University Hospital, Bergen, Norway; 4 Mohn Medical Imaging and Visualization (MMIV) Centre, Department of Radiology, Haukeland University Hospital, Bergen, Norway; 5 Department Strategy and Management, NHH Norwegian School of Economics, Bergen, Norway

**Keywords:** health guidance, large language models, diabetes, knowledge, helpfulness, empathy, Likert scale

## Abstract

Effective diabetes care relies on communication, patient empowerment, and lifestyle management. However, rising prevalence and workforce shortages challenge current care models. Large language models (LLMs) have the potential to support healthcare delivery by providing personalized health information. While prior studies show promising results, few have compared LLM-generated responses with those from healthcare professionals in chronic disease contexts, particularly from end-users' perspectives. This study compared GPT-4o and healthcare professional responses to diabetes-related questions, evaluating them on knowledge, helpfulness, and empathy. It also explored correlations between these qualities and differences based on participants' educational background. Using a cross-sectional experimental design, 1,810 evaluations were collected through an online questionnaire (November 2024–January 2025). Participants rated responses on 5-point Likert scales for knowledge, helpfulness, and empathy. For all metrics combined, GPT-4o received higher ratings in 46.7% of evaluations (95% CI: 28.8%–64.5%), while healthcare professionals were preferred in 23.3% (95% CI: 8.2%–38.5%). Participants with lower education levels rated GPT-4o significantly higher across all dimensions, while those with ≥4 years of higher education rated it higher for empathy and helpfulness. Quality measures were strongly correlated. Although differences were statistically significant, the observed effect sizes were small and should be interpreted as modest in practical terms. These findings assess perceived quality and accessibility of healthcare communication from end-user perspectives and suggest that LLMs may enhance the perceived quality and accessibility of healthcare communication, particularly among individuals with lower educational attainment. Further research is needed to determine their appropriate role in clinical practice, including objective assessment of clinical accuracy.

## 1 Introduction

Diabetes mellitus is a group of metabolic disorders characterized by elevated blood glucose levels, mainly due to impaired insulin secretion, reduced sensitivity, or a combination of both (American Diabetes Association Professional Practice Committee, 2025). It is among the leading causes of preventable mortality and morbidity worldwide (Aryal et al., 2023). Over the past decades, the global incidence and prevalence of diabetes have steadily increased, with recent estimates indicating that more than 800 million people are affected globally [(NCD Risk Factor Collaboration (NCD-RisC), 2024; World Health Organization, 2024a)]. The burden of diabetes extends beyond glycemic control, as it significantly increases the risk of a wide range of complications. These include cardiovascular diseases such as atherosclerosis, heart failure, and arrhythmia, as well as chronic kidney disease, diabetic retinopathy, neuropathy, and diabetic ketoacidosis (American Diabetes Association, 2025a,b). Effective diabetes management requires a multifaceted approach, including lifestyle modifications, regular blood glucose monitoring, pharmacological treatment, and the prevention and management of both hypoglycemia and hyperglycemia. Central to this is patient self-empowerment, which is closely linked to diabetes education and self-management strategies, and effective communication with healthcare professionals (Lambrinou et al., 2019). Beyond its clinical complications, diabetes imposes a considerable strain on the healthcare system. The rising incidence of the disease carries both social and economic consequences, with global healthcare expenditures reaching hundreds of billions of dollars annually (Chan et al., 2021). This growing demand for care, combined with a critical shortage in the health and care workforce (World Health Organization Regional Office for Europe, 2022), underscores the need for innovative solutions to support the management of this complex condition.

In recent years, the use of artificial intelligence (AI) in healthcare has expanded beyond diagnostics to encompass personalized patient engagement and empowerment. Virtual health assistants have been found to increase medical compliance (Reddy et al., 2019). Among the promising developments are large language models (LLMs), such as the Generative Pre-trained Transformer (GPT) family of models from OpenAI. These models represent a shift toward predictive, preventive, personalized, and participatory (P4) medicine by enabling tailored health guidance and enhancing patient autonomy (Sagner et al., 2017). By providing low-latency, adaptive responses to patient inquiries, they can support more effective self-management, enhance health literacy, and promote active participation in treatment decisions (Clusmann et al., 2023). Given these capabilities, LLMs may be particularly valuable in diabetes care, where long-term management relies on patient empowerment, adherence, and lifestyle changes (Denecke et al., 2024; Reddy, 2023; Chen et al., 2024; World Health Organization, 2024b).

By assisting both patients and caregivers, LLMs may enhance collaborative care models, making diabetes management more proactive and patient-centered. Despite this potential, their implementation in the field of medicine poses challenges regarding ethical and security concerns, bias in training data, and reliability (Denecke et al., 2024; World Health Organization, 2024b; Elendu et al., 2023). Addressing these challenges is critical to ensure that these models are not only practical but also equitable and safe for real-world applications (Reddy, 2023).

Previous research on the quality of LLM-generated healthcare responses has produced mixed findings. While several studies report promising results for various GPT-models (Ayers et al., 2023; Segal et al., 2024; Mork et al., 2025), others have found that physicians outperform these models in terms of accuracy (Arvidsson et al., 2024).

Most existing studies have focused on general medical knowledge or the models' ability to provide empathetic responses. However, few have systematically explored how patients and clinicians evaluate or experience responses from LLMs compared to those provided by human healthcare professionals in the context of chronic disease (Chen et al., 2024; Ayers et al., 2023). As (Moy et al. 2024) emphasize, investigating the patient's perspective is crucial for understanding how AI can be meaningfully integrated into clinical care.

This study aims to assess and compare responses from GPT-4o (OpenAI et al., 2024) and healthcare professionals to diabetes-related questions across three key dimensions: knowledge, helpfulness, and empathy, as perceived by both patients and healthcare professionals. In addition to the direct comparison, the study examines the relationship between these dimensions and factors such as question and answer length, evaluation time, and participants' educational background.

## 2 Materials and methods

### 2.1 Study design and aim

**Figure 1:**
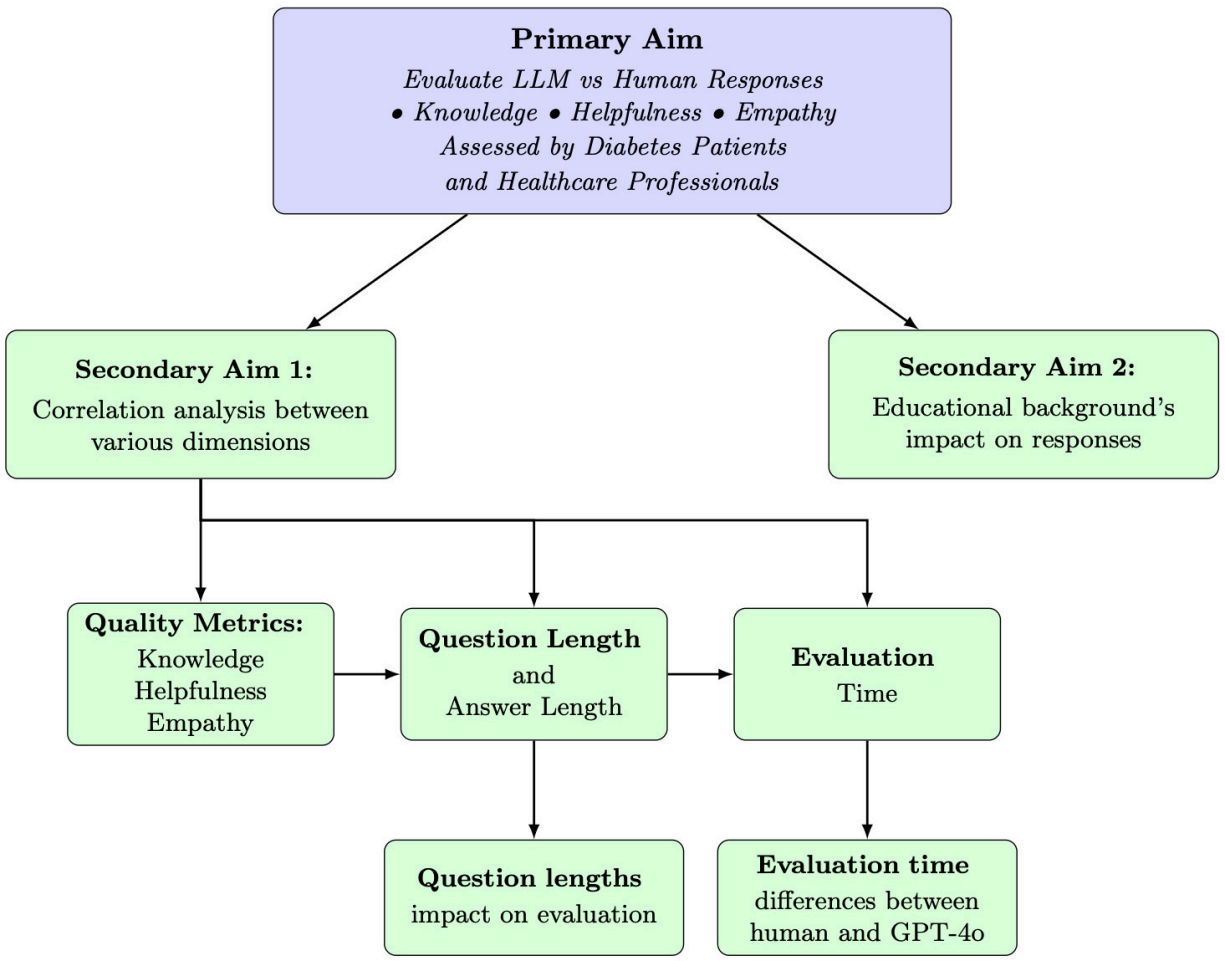
Overview of the study aims. The primary aim was to compare responses from GPT-4o and healthcare professionals to diabetes-related questions across three quality metrics: knowledge, helpfulness, and empathy, as evaluated by both patients and healthcare professionals. Secondary aim 1 explored correlations between quality metrics, question and answer length, and evaluation time. Here, evaluation time refers to the duration participants spent rating each response across the three dimensions. Secondary aim 2 examined how participants' educational background influenced response evaluations. Arrows represent analytical pathways and relationships between study components. LLM, large language models, represented in this study by GPT-4o, version 2024-08-06.

This study used a quantitative experimental, cross-sectional design to compare responses from GPT-4o (version 2024-08-06) and healthcare professionals to diabetes-related questions, as illustrated in Figure 1.

Data were collected between November 14, 2024, and January 31, 2025.

### 2.2 Materials and question selection

The questions used in this study were sourced from publicly available question-and-answer (Q&A) websites published by the Norwegian and Danish Diabetes Federations. To ensure broader topic coverage and international relevance, we included additional questions from comparable U.S.-based sources. The questions addressed a wide range of themes including lifestyle modifications, pharmacological treatment options, acute and chronic complications, psychological concerns, and clinical decision-making. The set of diabetes-related patient questions we included in the study comprised both concise, fact-based questions (e.g. Prompt 1) and more complex inquiries involving emotional and medical considerations (e.g. Prompt 2).

**Table:** 

**Patient inquiry:**

*Can diabetes affect sex life?*
**Healthcare professional response:**
Yes, diabetes can affect your sex life. It may impact you psychologically, making you feel less interested in sex or too tired.

**GPT-4o response:**
Yes, diabetes can affect your sex life. High blood sugar levels can damage nerves and blood vessels, which may lead to reduced sexual function. In men, this can result in erectile dysfunction, while women may experience vaginal dryness and decreased libido. Good blood sugar control, stress management, and discussing the issue with a healthcare professional can help improve the situation.


PROMPT 1. Example of a short patient-initiated question and corresponding answers generated by healthcare professional and GPT-4o. The responses illustrate differences in communication style, clinical depth, and comprehensiveness when addressing sensitive topics in diabetes care.

**Table:** 

**Patient inquiry:**
*Hi, I'm 50 years old and have had type 1 diabetes for 38 years. I'm in good shape, exercise regularly, don't smoke, and use an insulin pump. I'm also being treated with Levaxin (for hypothyroidism) and Candesartan (for high blood pressure). I've read about microneedling with Dermapen and would like to try it. One of the articles stated that it's contraindicated in people with diabetes. Is that true? If so, why?*

**Healthcare professional response:**
I've tried to find information about this, and it seems that most clinics specify that the concern mainly applies to poorly regulated diabetes. Therefore, it should generally be safe if your diabetes is well controlled and your HbA1c is within the reference range. I believe the primary concern is the risk of slow wound healing, as the Dermapen creates small wounds in the skin. If you feel unsure, it's a good idea to consult a dermatologist or doctor to clarify this before undergoing this type of treatment.

**GPT-4o response:**

Hi, Microneedling, including the use of Dermapen, can pose a potential risk for individuals with diabetes, particularly due to the increased risk of infection and delayed wound healing. Diabetes can affect blood flow and the immune system, which may reduce the body's ability to fight infections and effectively repair skin damage. It is important to discuss this type of treatment with your endocrinologist or general practitioner before proceeding. They can provide guidance based on your specific medical history and current health status. Make sure to keep your blood sugar well controlled, and ensure proper hygiene and follow-up if you choose to go ahead with microneedling. Best regards


PROMPT 2. Example of a long patient-initiated question and corresponding answers generated by a healthcare professional and GPT-4o. The responses illustrate differences in communication style, clinical depth, and comprehensiveness when addressing sensitive topics in diabetes care.

All study materials were presented to participants in Norwegian. Questions originally sourced from Danish and English platforms were translated into Norwegian for use in the study. Norwegian questions and responses presented in this manuscript have been translated into English for publication. Clinical reference values were adapted to align with Norwegian national clinical practice guidelines. All materials were anonymized, and any personally identifiable information was removed.

Inclusion criteria comprised patient-initiated questions about diabetes management, treatment, lifestyle, or complications. Exclusion criteria included questions exceeding 300 words (to ensure mobile readability and reduce participant fatigue), patient inquiries that did not contain a clear question (such as general feedback or comments), and content with personally identifiable information. Based on these criteria, a total of 113 patient questions were included in the final study materials.

Healthcare professional responses were written by clinicians (physicians, nurses, or diabetes educators) contributing to these public Q&A platforms. Individual credentials were not verified as responses were sourced from established diabetes federation websites. Each question had one corresponding response written by a healthcare professional on these platforms. No combining or synthesis of multiple professional responses was performed; each represented one clinician's original answer to one patient question.

GPT-4o responses were generated using standardized prompting (System Message 1) via Azure OpenAI API in September 2024. Each of these 113 selected questions was posed to both healthcare professionals and GPT-4o. The resulting responses were compiled into a structured file format to ensure a standardized format for subsequent evaluation. Question lengths ranged from 3 to 199 words (mean = 40.6, SD = 43.8, median = 35). GPT-4o responses ranged from 36 to 331 words (mean = 121.6, SD = 69.8, median = 96), while responses from healthcare professionals ranged from 19 to 286 words (mean = 114.9, SD = 65.2, median = 101).

**Table:** 

You are to assume the role of a highly experienced medical doctor or healthcare professional with extensive knowledge of diabetes and the issues patients face.

**Instructions:**
• Provide all responses in well-written Norwegian.
• Use clear and compassionate language appropriate for patients seeking medical advice.
• Ensure that information is accurate and up-to-date based on current medical guidelines in Norway.
• Keep responses focused on the question, avoiding unnecessary details.
• Keep your responses short and concise.
• Do not sign your responses.


SYSTEM MESSAGE 1. Specification of the system prompt used to generate standardized simulated healthcare professional responses in Norwegian. The prompt was designed to incorporate key elements of medical communication, including professional role, language requirements, clinical accuracy, and conciseness. It instructed GPT-4o to base its responses on Norwegian medical guidelines for diabetes care; however, without search capabilities, the model could only rely on its training data, which may be incomplete or outdated.

### 2.3 Participants recruitment and characteristics

**Figure 2:**
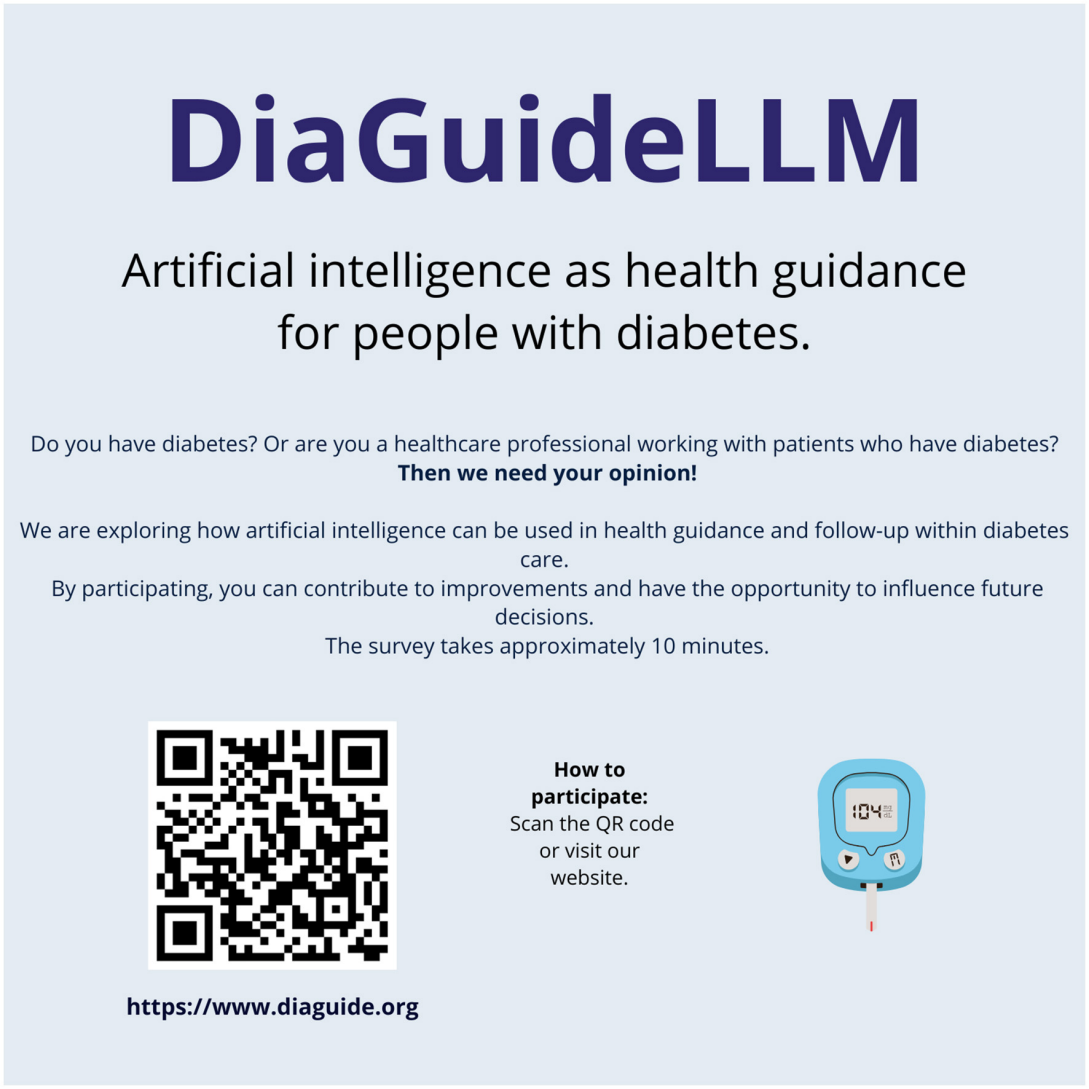
This translated version reflects the original Norwegian-language flier used to recruit study participants. The flier invited both individuals living with diabetes and healthcare professionals to take part in a brief online survey, estimated to take approximately 10 min to complete. It was distributed at local hospitals and general practitioners offices in Bergen, Norway, as well as digitally in relevant Facebook groups.

Participants from the two target groups–individuals with diabetes and healthcare professionals– were recruited through a combination of online and offline strategies to achieve a diverse and representative sample. A recruitment flier containing key information about the study, along with a QR code and a direct link to the questionnaire, is illustrated in Figure 2.

It was distributed digitally in relevant Facebook groups aimed at people with diabetes and healthcare professionals, including medical students. To engage participants beyond digital platforms, physical copies of the flier were posted in two local hospitals and distributed to general practitioners' offices in the Bergen area.

A demographic overview of the study participants is provided in Table 1.

**Table 1:** Demographic and professional characteristics of study participants.

**Characteristic**	***n***	**%**
**Total study population**		
	273	100.0
**Demographics**		
**Age, years**		
18–29^a^	45	16.5
30–39	44	16.1
40–49	74	27.1
50–59	70	25.6
≥ 60	40	14.7
**Gender**		
Female	218	79.9
Male	53	19.4
Not disclosed	2	0.7
**Clinical status** ^b^		
Persons with diabetes (non-healthcare)	175	64.1
Healthcare professionals (non-diabetic)	33	12.1
Healthcare professionals with diabetes	48	17.6
General population	17	6.2
**Educational background**		
Primary and lower secondary education	76	27.8
**Higher education**		
Higher education 1–3 years	89	32.6
Master level^c^	46	16.8
Advanced graduate studies^d^	41	15.0
Doctoral degree	14	5.1
Not disclosed	7	2.6

^a^Includes 4 participants aged ≤ 19 years.

^b^Healthcare professionals included physicians (n = 18), nurses (n = 26), medical students (n = 7), and other healthcare workers (n = 29).

^c^4–5 years of higher education.

^d^6+ years of higher education, excluding doctoral degrees.

**Figure 3:**
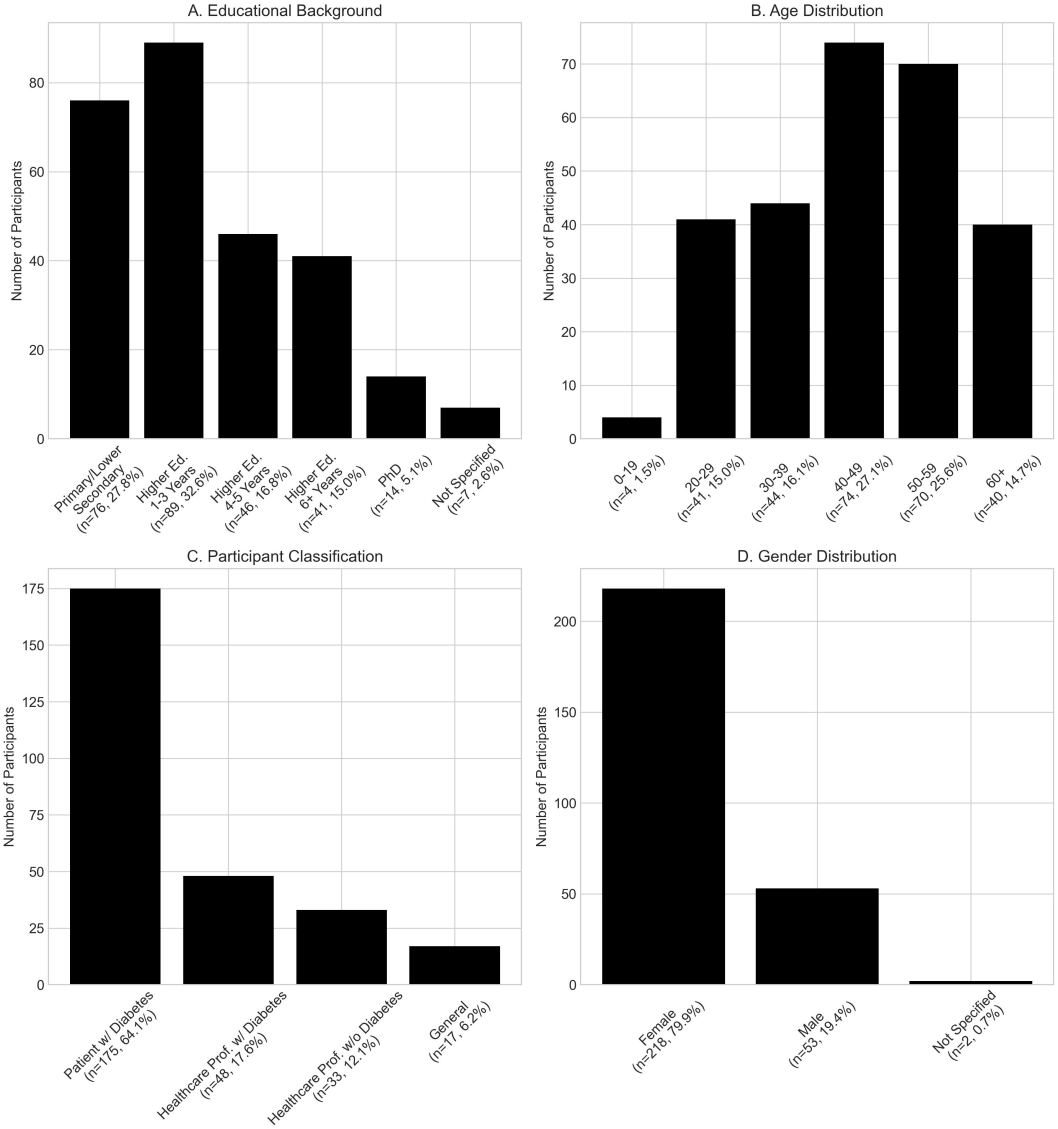
Demographic characteristics of study participants (*n* = 273). Distribution of **(A)** educational background, **(B)** age groups, **(C)** participant classification between healthcare professionals and diabetes patients, and **(D)** gender. Values represent absolute numbers and percentages of total study population. The study sample was predominantly female (79.9%), aged 40–59 years (52.7%), and mainly diabetes patients (64.1%).

A more granular graphical presentation is given in Figure 3.

### 2.4 Data collection procedures

#### 2.4.1 GPT-4o responses

Responses were generated in September of 2024 using version 2024-08-06 of GPT-4o. No additional context or instructions were provided to maintain consistency across responses. All interactions with the model were documented, and the generated responses were stored in the same structured file format as those from healthcare professionals.

#### 2.4.2 Survey implementation

The evaluation survey was administered through a custom-built web platform optimized for both desktop and mobile devices (available at https://github.com/alu042/HumanVsRobot). On the introductory page, participants were informed about the purpose of the study and assured that their responses would remain anonymous. Proceeding beyond this page was considered as providing informed consent for the use of their responses in research. Participants were then asked to provide demographic and background information, including age group, gender, diabetes status, healthcare professional status, education level, and prior participation in the survey. These characteristics are summarized in Table 1. The variables were collected to enable analysis of potential associations between participant characteristics and their evaluation of responses.

Each participant was presented with 10 questions and their corresponding answers. For each question, participants evaluated only one response, either from a healthcare professional or from GPT-4o. Response assignment was randomized, with question selection weighted to prioritize responses with fewer prior evaluations, ensuring balanced distribution of ratings across all materials. Participants were not shown paired comparisons; each evaluation was conducted independently. Random assignment resulted in balanced evaluation distribution, with 890 GPT-4o evaluations and 920 healthcare professional evaluations. This minor imbalance (3.3% difference) does not compromise the validity of comparisons, as the statistical methods applied account for differing sample sizes.

Participants assessed each answer using a 5-point Likert scale, chosen for accessibility and to reduce participant burden, following common practice in healthcare communication research (Sullivan and Artino, 2013), across three dimensions:

1. Knowledge (“assess the accuracy and relevance of the information provided in the answer.”)
2. Helpfulness (“evaluate to what extent the answer provides useful and practical advice or support that can help the person asking.”)
3. Empathy (“judge the answer's ability to show understanding for the patient's feelings and concerns.”)

The scale ranged from 1 (“very poor”), 2 (“poor”), 3 (“neutral”), 4 (“good”) to 5 (“very good”). This format follows (Marsh, 1982), who used a 5-point Likert-type scale from “very poor” to “very good” to assess teaching quality. Response times were recorded for each answer evaluation.

### 2.5 Statistical analysis

The D'Agostino-Pearson omnibus test was used to assess the normality of the data. As the data deviated from a normal distribution, non-parametric statistical methods were applied for all subsequent analyses. Analyses were performed using Python (version 3.11.11) with *Pandas* and *SciPy* libraries. The Likert scale data was treated as either categorical or ordinal, depending on the specific variable being analyzed. A significance threshold was set to *p* < 0.05. Responses with incomplete evaluation were excluded from the corresponding analyses. Note that this study did not employ multiple raters evaluating identical responses. Each response was evaluated by different participants (mean = 16.02 ratings per question from independent evaluators). The design focused on aggregating independent evaluations across a diverse sample rather than measuring agreement between raters on identical content. Therefore, inter-rater reliability metrics such as Cohen's kappa or intraclass correlation coefficient (ICC) are not applicable to this design.

#### 2.5.1 Primary analysis

To compare participant evaluations of responses generated by GPT-4o and healthcare professionals, a frequentist statistical approach was employed using the Chi-square test of independence. Effect sizes were calculated using Cramér's *V*, a measure of association for categorical variables derived from the Chi-square statistic. For degrees of freedom (df) = 4, values of Cramér's V were interpreted as small (< 0.2), medium (< 0.3), or large (≥0.3) effects. The Wilson score interval was used to compute confidence intervals for the percentage distribution of preference. To account for multiple comparisons across the three evaluated dimensions, a Bonferroni correction was applied, adjusting the significance threshold to α = 0.017. This conservative approach was chosen to reduce the risk of Type I errors. Analyses were repeated within the diabetes patient subgroup using the same statistical procedures.

#### 2.5.2 Secondary analysis

The Spearman's rank correlation coefficient (ρ) was used to examine associations among the three evaluation dimensions: knowledge, empathy, helpfulness, as well as between these dimensions and objective characteristics of the responses, including length of question and answer and evaluation time. Correlation strength was interpreted according to the following thresholds: “very weak” (ρ ≤ 0.19), “weak” (0.20 ≤ ρ ≤ 0.39), “moderate” (0.40 ≤ ρ ≤ 0.59), “strong” (0.60 ≤ ρ ≤ 0.79) and “very strong” (ρ≥0.80). A Bonferroni correction was again applied, adjusting the significance threshold to α < 0.003 to adjust for multiple testing.

To ensure balanced group sizes while preserving meaningful distinctions in educational level, some educational categories were merged. This resulted in three categories; primary and lower secondary education, higher education 1–3 years and higher education ≥4 years. The Kruskal–Wallis test was conducted to assess group differences in response evaluation across the three quality metrics (knowledge, helpfulness, and empathy). When significant differences were detected, *post hoc* pairwise comparisons were performed using Mann–Whitney *U* test. To control for multiple testing across the nine comparisons, a Bonferroni correction was applied, setting the adjusted significance threshold to α = 0.006. Effect sizes were calculated using Cliff's delta (δ), with the following interpretation: “negligible” (δ < 0.147), “small” (0.147 ≤ δ < 0.33), “medium” (0.33 ≤ δ < 0.474), and “large” (δ≥0.474).

To explore the relationship between question length and evaluation scores, questions were classified as either “short” or “long,” based on the median word count (35 words). Separate Mann–Whitney *U*-tests were conducted for each quality dimension and question length. Cliff's delta was used to quantify effect sizes. Differences in scores between groups were reported both as percentage change and as raw point differences on the Likert scale. For comparisons of evaluation time, the Mann–Whitney *U*-test was again used, with Cliff's delta for effect size estimation. Measures of central tendency were reported as median values along with interquartile range (IQR).

#### 2.5.3 Power analysis

A *post-hoc* power analysis was conducted to assess the study's ability to detect the observed differences. Power calculations were based on effect sizes derived from the observed data and performed using the *statsmodels* package (version 0.14.2) in Python. The analysis indicated sufficient power (>80%) for the majority of comparisons. However, two exceptions were identified:

First, for evaluation time, the observed effect size was small (Cohen's *d* = −0.066; LLM group: *n* = 890, human group: *n* = 920), resulting in an achieved power of only 29.2%. The required sample size per group to achieve 80% power for this effect was estimated to be 3, 500, which exceeded the available sample size.

Second, for knowledge ratings within the subgroup of participants with 1–3 years of higher education, the observed effect size was also small (Cohen's *d* = 0.121; LLM: *n* = 304; human: *n* = 329), yielding an achieved power of 33.1%. The required sample size per group for 80% power in this comparison was 1, 027.

These results suggest that, while the study was adequately powered for most comparisons, caution is warranted when interpreting results for evaluation time and knowledge ratings in the 1–3 years higher education subgroup due to limited statistical power.

### 2.6 Ethical considerations

The project was reviewed by the Regional Committees for Medical and Healthcare Research Ethics, REK (https://www.forskningsetikk.no/en/about-us/our-committees-and-commission/rek). REK concluded that the study was not subject to mandatory ethical review (“ikke fremleggingspliktig”) and therefore did not require ethical review or approval. This decision was based on the expectation that the study would not generate new knowledge about health or disease. It was registered with the Data Protection Commissioner and registered in RETTE (https://rette.app.uib.no), a system for managing personal data in research projects at the University of Bergen.

## 3 Results

### 3.1 Descriptive statistics

A total of 1, 810 response ratings were collected, comprising 890 evaluations of GPT-4o responses and 920 evaluations of responses from healthcare professionals. In total, 273 participants took part in the study, evaluating 113 unique questions and 203 corresponding answers (LLM: *n* = 103 human: *n* = 100). On average, each participant contributed 6.6 ratings (*SD* = 3.96), with a median of 10.0 ratings. The mean number of ratings per question was 16.02 (*SD* = 12.57). No participants (0.0%) completed the survey more than once.

### 3.2 Main findings

For the three metrics combined, responses generated by GPT-4o were preferred in 46.7% of evaluations (95% CI: 28.8%–64.5%), compared to 23.3% (95% CI: 8.2%–38.5%) for responses from healthcare professionals. In 30.0% of cases (95% CI: 13.6.%–46.4%), the responses were rated equally.

Chi-square analyses revealed significant differences between GPT-4o and healthcare professional responses across all quality metrics. Although the differences were statistically significant, effect sizes were small in all cases:

- Knowledge: χ^2^(4) = 17.66, *p* = 0.00144, Cramer's *V* = 0.099, (95*%CI*:0.061 − 0.153) (significant after Bonferroni correction, α = 0.017)
- Helpfulness: χ^2^(4) = 24.25, *p* < 0.001, *V* = 0.116, (95*%CI*:0.077 − 0.165) (significant after Bonferroni correction, α = 0.017)
- Empathy: χ^2^(4) = 23.79, *p* < 0.001, *V* = 0.115, (95*%CI*:0.077 − 0.165) (significant after Bonferroni correction, α = 0.017)

**Figure 4:**
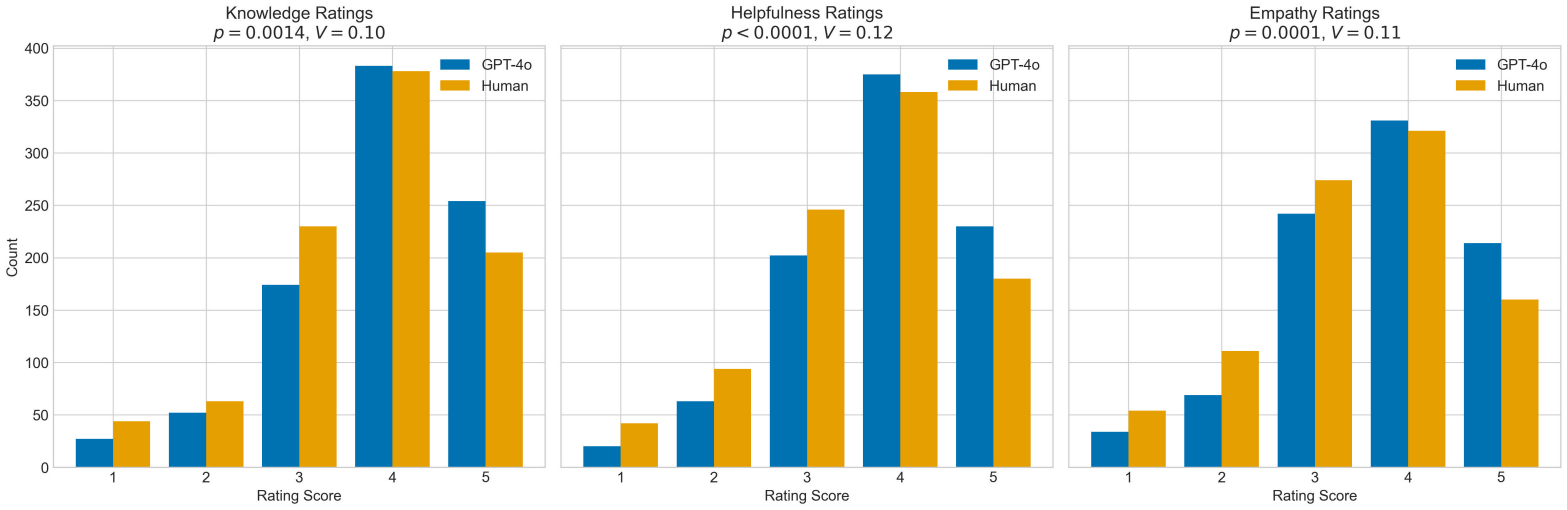
Distribution of knowledge, helpfulness, and empathy ratings: comparison between GPT-4o and healthcare professionals. Distribution of ratings (1–5 scale) comparing GPT-4o (blue) and healthcare professionals (orange) across three quality dimensions. All differences were statistically significant with small effect sizes (Knowledge: *p* = 0.0014, Cramer's *V* = 0.10; Helpfulness: *p* ≪ 0.001, *V* = 0.12; Empathy: *p* ≪ 0.001, *V* = 0.11). Both groups show right-skewed distributions with modal ratings of four across all dimensions.

These results are illustrated in Figure 4. After applying a Bonferroni correction (α = 0.017) for multiple comparisons, the differences remained statistically significant (*p*≲0.001 for all three dimensions).

GPT-4o responses were more frequently rated with the highest score (Likert = 5), with an increase of 6.7 % compared to healthcare professionals (GPT-4o: 24.04%, Human: 17.39%). In contrast, healthcare professional responses received higher proportions of ratings in the lower categories for the empathy dimension (Empathy ratings 1–3: GPT-4o: 38.76%, Human: 47.72%). Subgroup analysis restricted to participants with diabetes showed a similar pattern to the main analysis, i.e., Diabetes subgroup: Empathy ratings = 5: GPT-4o: 23.87%, Human: 17.26%; Empathy ratings 1–3: GPT-4o: 39.47%, Human: 48.34%.

### 3.3 Additional findings

**Figure 5:**
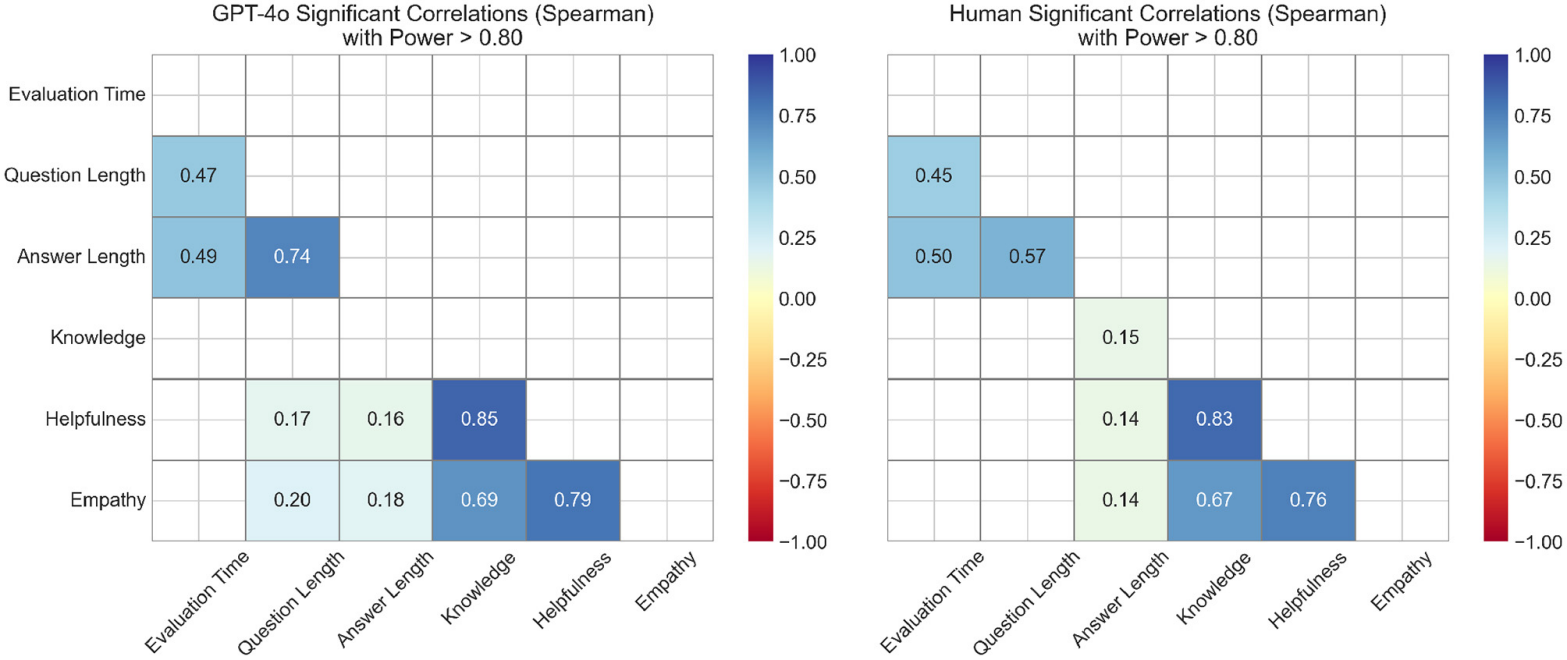
Correlation analysis presenting Spearman's ρ-values, only showing results with both statistically significant after Bonferroni correction (α < 0.003) and sufficient power (>80%). Correlation strength was classified by ρ as “very weak” ( ≤ 0.19), “weak” (0.20–0.39), “moderate” (0.40–0.59), “strong” (0.60–0.79) and “very strong” (≥0.80).

Figure 5 presents the results of the correlation analyses between evaluation time, question length, answer length, knowledge, helpfulness, and empathy. Only correlations that were statistically significant after Bonferroni correction (α < 0.003) and demonstrated sufficient power (>80%) are reported.

The strongest correlations were observed among the quality metrics, particularly between knowledge and helpfulness. A “strong” association was also found between question length and answer length in GPT-4o responses. “Moderate” correlations were identified between question length and evaluation time, as well as between answer length and evaluation time, in both the GPT-4o and the healthcare professional groups.

The absence of correlation between answer length and knowledge ratings for GPT-4o may reflect its consistent response structure regardless of content complexity, whereas human responses show more variability in length based on the depth of information provided. The strong correlation between knowledge and helpfulness (ρ>0.80) suggests participants perceived well-informed responses as inherently more useful. While these dimensions are conceptually distinct (knowledge reflects accuracy and completeness while helpfulness reflects practical utility), participants may have difficulty separating these constructs in practice, as acknowledged in our limitations.

For “long” questions (more than 35 words), GPT-4o responses received higher empathy scores, with an increase of 0.40 points from “short” to “long” questions (3.55 to 3.94), corresponding to an 11.2% improvement. Compared to responses from human healthcare professionals, GPT-4o demonstrated a greater gain in empathy scores between “short” and “long” questions (0.40 vs. 0.18 points), with a small but statistically significant effect size (Cliff's δ = 0.213, *p* < 0.001).

Participants also spent slightly less time evaluating GPT-4o responses than those written by healthcare professionals. The median evaluation time for GPT-4o was 56.9 s (IQR: 36.1-81.9 s), compared to 59.0 s for healthcare professional responses (IQR: 40.7-88.5 s; *p* = 0.0813, Cliff's δ = −0.074). The mean evaluation times (GPT-4o: 70.9 s, Human: 124.3 s) exceeded their respective medians, indicating a right-skewed distribution, particularly for human responses, due to the presence of some exceptionally time-consuming evaluations.

Subgroup analyses by age, gender, and clinical status revealed patterns consistent with overall findings, with no significant interactions detected after Bonferroni correction.

**Figure 6:**
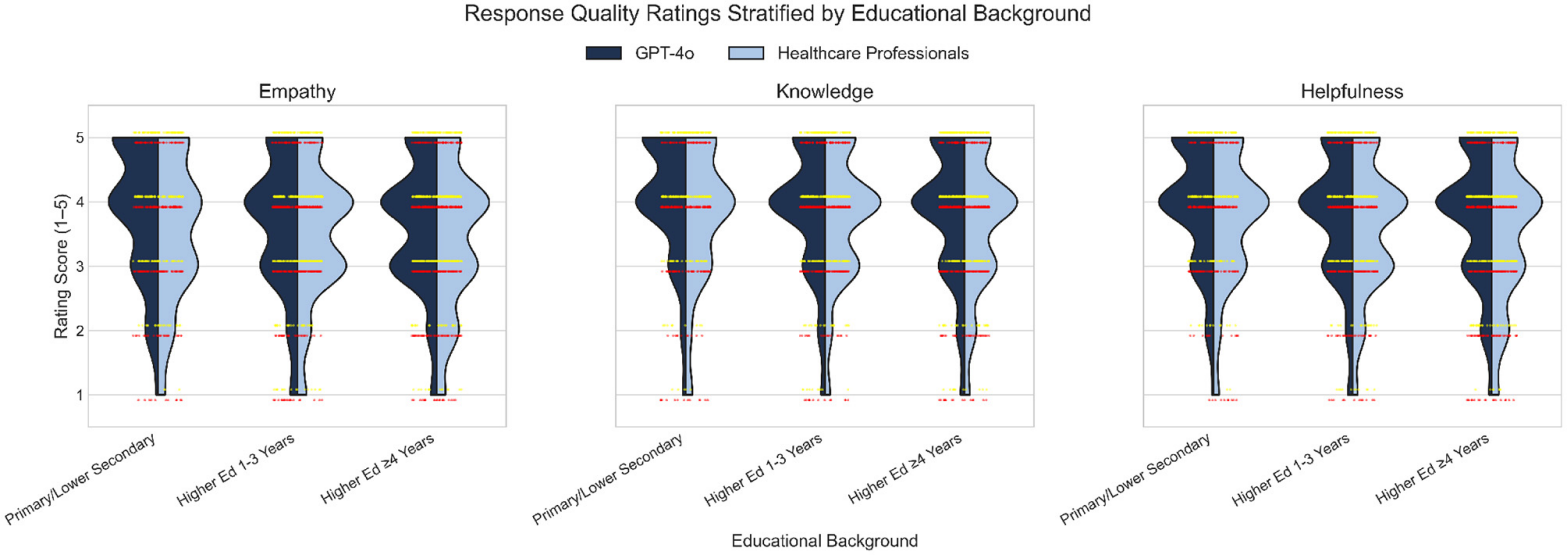
Distribution of response quality ratings for GPT-4o (dark blue) and healthcare professionals (light blue) across three educational backgrounds and three evaluation dimensions (empathy, knowledge, and helpfulness). Split violin plots display the full distribution of ratings (1–5 Likert scale) for each group, with the left half representing GPT-4o and the right half representing healthcare professionals. Individual ratings are overlaid as jittered dots, with yellow points indicating GPT-4o evaluations and red points indicating healthcare professional evaluations, horizontally offset for clarity.

Figure 6 illustrates the distribution of response quality ratings for GPT-4o and healthcare professionals across three educational backgrounds and three evaluation dimensions. Across all educational groups and metrics, GPT-4o responses consistently received higher ratings than those of healthcare professionals. This difference was most pronounced among participants with primary and lower secondary education, where the distributions for GPT-4o were clearly shifted toward higher scores compared to those for healthcare professionals. The split violin plots reveal that, for all groups, the majority of ratings clustered at the upper end of the scale, but the density of high ratings (scores of 4 and 5) was greater for GPT-4o. The overlaid jittered points further highlight the concentration of high scores for GPT-4o (yellow) and the relatively broader spread of ratings for healthcare professionals (red), particularly in the lower education group. These findings indicate that GPT-4o responses were perceived as higher quality across all educational backgrounds, with the largest advantage observed among participants with lower educational attainment.

The most pronounced difference was observed in empathy scores, where GPT-4o responses received higher ratings than healthcare professional responses by a 10.1% difference in mean [GPT-4o: Mean = 3.91 vs. Human: Mean = 3.55; Cliff's δ = 0.182 (95% CI: 0.074–0.290), *p* = 0.0006]. Similarly, knowledge ratings were 7.9% higher [GPT-4o: Mean = 4.07 vs. Human: Mean = 3.77; δ = 0.162 (95% CI: 0.054–0.270), *p* = 0.0018], and helpfulness ratings were 7.2% higher [GPT-4o: Mean = 4.00 vs. Human: Mean = 3.73; δ = 0.147 (95% CI: 0.039–0.255), *p* = 0.0047].

Among participants with advanced higher education (≥4 years), GPT-4o responses were also rated higher for empathy [7.0% difference in mean; δ = 0.117 (95% CI: 0.032–0.203), *p* = 0.0051] and helpfulness [7.4% difference in mean; δ = 0.128 (95% CI: 0.042–0.213), *p* = 0.0022]. No significant differences were found in the group with 1–3 years of higher education (*p*>0.01) with differences in mean < 5.1% for GPT-4o responses vs. Human for all three measures. Across all education groups, effect sizes remained small (all δ < 0.33), suggesting modest but consistent advantages in favor of GPT-4o responses.

## 4 Discussion

This study contributes to a growing body of research exploring the potential of LLMs in healthcare communication. Our findings show that GPT-4o consistently received higher ratings than healthcare professionals in perceived knowledge, empathy, and helpfulness, with the largest differences observed in empathy and helpfulness. Although the differences were statistically significant, effect sizes were small and median scores across all dimensions were uniformly high (median = 4.0). As such, the practical or clinical significance of these differences may be limited. Higher subjective ratings do not necessarily translate to better clinical outcomes or clinical accuracy, which are fundamentally different constructs.

The present findings are consistent with (Mork et al. 2025), who reported that GPT-4 responses were rated higher than those of physicians in terms of empathy, knowledge, and helpfulness. Similarly, (Ayers et al. 2023) found that GPT-3.5-generated responses were perceived as more empathetic and of higher quality compared to those of physicians. While both studies reported larger statistical differences than ours, this discrepancy may be attributed to methodological differences, particularly our inclusion of both patient and healthcare professional evaluators. By capturing diverse perspectives, this study's approach may have yielded a broader and potentially more representative assessment of response quality. This supports the importance of including patient voices in future AI evaluation studies (Moy et al., 2024).

(Arvidsson et al. 2024) compared GPT-4's performance to general doctors and top-tier specialists, finding that GPT-4 underperformed compared to human doctors. This discrepancy can possibly be attributed to methodological differences, as they focused more on the clinical accuracy and depth of medical knowledge within responses. These differences illustrate the importance of carefully considering the context-dependent strengths and limitations of large language models within healthcare communication. (Chen et al. 2024) compared manual responses, LLM-generated drafts, and LLM-assisted answers. They found that a small proportion of unedited LLM-drafts posed a potential risk of severe harm or even death, primarily due to incorrect evaluation of the acuity and inappropriate recommendations. Given the known risks of LLMs in healthcare including potential for misinformation and inappropriate recommendations, the modest perceived advantages in subjective ratings observed in our study do not, by themselves, establish that direct patient-facing deployment is warranted without clinical oversight. The small effect sizes further emphasize that practical clinical significance may be limited despite statistical significance. However, the researchers suggest that when used to assist rather than replace clinicians, LLMs may offer a “best-of-both-worlds” scenario; reducing workload on physicians and improving the informative and educational value of responses (Chen et al., 2024). AI-assisted replies were found to improve consistency across physicians and maintain overall response quality, while also revealing potential risks such as over-reliance on AI-generated assessments. In a related study, a knowledge-infused, specialized LLM outperformed GPT-4 regarding diabetes related questions, such as those concerning daily meal choices (Abbasian et al., 2024). AI-powered dietitians have also shown potential in assisting diabetes management through image recognition and personalized dietary recommendations (Sun et al., 2023). Together with our results, these findings suggest that LLMs have the potential to enhance healthcare communication, provided that clinical oversight remains a critical safeguard against unintended consequences.

To better understand our findings, we explored several potential correlations between the quality dimensions. A strong association was found between perceived knowledge and helpfulness, indicating that participants viewed well-informed responses as more useful. This suggests a general perception that knowledge and competence are essential components of high-quality healthcare. Similarly, empathy was moderately associated with helpfulness, highlighting the importance of emotional tone in perceived response quality. Note that empathy is also acknowledged to have a positive impact on health outcomes (Decety, 2020). Comparable correlation patterns were observed for both GPT-4o and human responses, indicating similar internal consistency across groups.

Structural patterns in the data were also identified. For GPT-4o, a strong association between question length and response length was observed, reflecting its consistent response structure. Healthcare responses showed greater variability, likely due to individuals' writing styles. Importantly, GPT-4o was explicitly instructed to keep responses “short and concise,” whereas healthcare professionals received no formatting instructions. This discrepancy may have influenced the observed differences and introduced a methodological bias.

Participants' educational background appeared to influence evaluation outcomes. Participants with either primary/lower secondary education or ≥ 4 years of higher education rated GPT-4o responses significantly higher than those of healthcare professionals. No significant differences were observed in the 1–3 years education group after Bonferroni correction, possibly due to limited statistical power. The higher ratings from participants with lower education may reflect GPT-4o's use of simplified language and patient-centered framing, in line with its system prompts. Alternative explanations warrant consideration. The simplified language and patient-centered framing of GPT-4o responses may genuinely improve accessibility for participants with lower educational attainment. However, if stylistic features (e.g., formatting, tone, or structure) allowed participants to implicitly infer the source of the response, differences in familiarity with or attitudes toward technology-generated content across educational groups could have influenced ratings independently of clinical quality. Distinguishing between these explanations requires future studies incorporating objective quality metrics alongside subjective ratings.

Conversely, healthcare professional responses, despite potentially being richer in clinical detail, may have been perceived as less accessible. These findings highlight the importance of tailoring communication to different patient populations. Health literacy is closely associated with education and is a known predictor of poorer health outcomes and higher mortality (Friis et al., 2016; Smith et al., 2018). Prior research has shown that cognitive, emotional, and psychological factors influence patients' ability to engage with and act on health information (Gonzalez et al., 2016). Since LLMs generate information based on a given prompt, users can influence and personalize answers according to their existing knowledge. Thus, such tools have the potential to help overcome barriers to health literacy and empower patients with accessible knowledge (Denecke et al., 2024). The present analysis focused on perceived quality rather than objective measures of response accuracy or educational effectiveness. Future studies should investigate whether AI-driven communication strategies can enhance patient adherence and ultimately improve diabetes management across diverse educational backgrounds.

### 4.1 Ethical considerations

The integration of large language models into healthcare offers diverse opportunities, including personalized education, patient empowerment, and improved accessibility to health information (Clusmann et al., 2023). However, these potential benefits are accompanied by critical concerns that must be addressed to mitigate potential risks (Wang et al., 2023). Key challenges include data privacy and security, algorithm bias and fairness, accountability and transparency, and the broader implications for clinical responsibility and professional integrity (Elendu et al., 2023). From a regulatory standpoint, ensuring legal responsibility, data protection, and compliance is essential. Within the clinical context, the deployment of LLMs must also consider their impact on the physician-patient relationship, the humanistic aspects of care, and the preservation of professional integrity (Wang et al., 2023).

Even with ethical frameworks in place, significant risks remain. Among the most pressing concerns are LLMs' potential to generate misinformation, fabricate sources, plagiarize or “hallucinate”—that is, to produce inaccurate or misleading content in a persuasive tone. In healthcare, these issues become particularly critical (Reddy, 2023; Sng et al., 2023). (Shiferaw et al. 2024) demonstrated that ChatGPT's responses to identical medical queries could vary substantially in content and reliability, and sometimes included references that did not exist in the academic literature. Such behavior may mislead users, particularly those with limited medical knowledge, reinforcing the need for validation mechanisms to ensure the content's accuracy in health applications. Clinical oversight remains critical, and perceived quality differs fundamentally from objective accuracy and safety.

The ethical development of AI in healthcare is an evolving process that demands transparency, fairness, and multi-stakeholder collaboration. As emphasized by international bodies such as the World Health Organization (WHO), ethical implementation must prioritize patient safety and wellbeing (World Health Organization, 2024b). The European Union's AI Act and Norway's national AI strategy for health services (2024–2025) reflect growing recognition of the need for robust regulatory oversight (European Union, 2024; Helsedirektoratet, 2025). Ongoing reflection and adaptation will be critical to integrating LLMs in ways that enhance trust, reduce harm, and ensure equitable access to high-quality care (Elendu et al., 2023).

### 4.2 Strengths and limitations

#### 4.2.1 Strengths

This study offers a unique contribution to the literature by systematically comparing responses from GPT-4o and human healthcare professionals to diabetes-related inquiries, evaluated by both patients and healthcare providers. The dual perspective adds validity and captures a broader understanding of how LLMs are perceived in a real-world health communication context. A structured evaluation framework, based on empathy, knowledge, and helpfulness, enabled standardized and replicable assessments. The dataset is based on publicly available Q&A content, and the survey design allows for full reproducibility. Diverse questions and a combined online and offline recruitment strategy enhanced the realism of interactions and the generalizability of findings.

#### 4.2.2 Limitations

This study was conducted in a cross-sectional format, using static Q&A rather than real-time interaction. Therefore, the results may not fully reflect the dynamics of live healthcare consultations, where factors such as trust, tone, urgency, non-verbal cues, trust-building, clarification requests, and conversational adaptation shape patient perception and decision-making. These dynamic factors cannot be captured in our static design.

The sample was predominantly female (79.9%), which limits generalizability. Research suggests that perceptions of communication quality and empathy may differ across genders. Therefore, findings may not fully represent responses from a gender-balanced population, and caution is warranted when extrapolating to male patients or mixed-gender populations. Participant recruitment was primarily digital, potentially skewing the sample toward individuals with higher digital literacy. The demographic skew (79.9% female, 52.7% ages 40–59, only 6.2% general population without diabetes) limiting broader representativeness. The underrepresentation of non-patient perspectives limits insights into broader population perceptions.

The public Q&A used may overrepresent common or easily answered queries, while some responses received relatively few evaluations, reducing robustness in subgroup analyses.

Although the survey was designed to minimize bias, participants were not blinded to stylistic differences between responses from healthcare professionals and GPT-4o. The survey design did not explicitly identify responses as LLM or human-generated. However, stylistic differences may have been apparent to participants. The LLM's occasional use of bullet points and its concise formatting may have influenced evaluations independent of content quality, introducing methodological bias that may overestimate GPT-4o advantages. This represents a significant limitation affecting interpretation of results.

Translation from Danish and English sources into Norwegian may have altered tone, nuance, and empathy expression, potentially affecting empathy ratings and introducing cultural bias.

Moreover, participants may have difficulties distinguishing between “knowledge” and “helpfulness,” introducing construct overlap.

Statistical limitations include the use of frequentist approaches that treat Likert data as categorical rather than ordinal. While the Chi-square test is appropriate for this design, ordinal regression or Bayesian approaches might better model rating scale complexity in future work. Additionally, participant clustering and repeat evaluations were not accounted for with hierarchical models, which may introduce dependency bias.

The study did not verify healthcare professionals' identity, credentials, or clinical experience levels. Variability in provider expertise may have affected comparison fairness and introduced unknown variability in baseline quality. Responses represent real-world healthcare communication as it exists on public platforms. It also lacked semantic or linguistic analysis of responses. Some participants reported difficulty evaluating diabetes types they were unfamiliar with, potentially introducing variability. Furthermore, comments such as “I learned a lot” suggest that participants may have engaged with the material educationally rather than to evaluate.

While the findings were statistically significant, the observed effect sizes were small, and should be interpreted as modest in practical terms. One possible explanation is the limited sensitivity of the Likert scale, which may reduce the ability to detect subtle differences and thereby underestimate the true effect. The high median ratings (4.0 across all dimensions) suggest potential ceiling effects, indicating that the 5-point scale may have limited granularity for detecting nuanced quality differences. This represents a measurement limitation that should be addressed in future studies. Although the overall sample size was substantial, segmenting the sample into subgroups and response types likely reduced statistical power for more granular comparisons. A trend toward shorter evaluation times for GPT-4o responses was observed; however, the current sample size lacked sufficient statistical power to confirm this effect. Nonetheless, the pattern may reflect a genuine difference and should be explored in future studies.

The study did not include systematic assessment of clinical accuracy or safety of responses. The study assessed perceived quality rather than clinical accuracy or educational effectiveness, which are fundamentally different constructs. Participants may have been misled by confidently stated but potentially inaccurate information. Validation of content accuracy against diabetes guidelines is essential before clinical implementation.

The study was conducted entirely in Norwegian, using Norwegian, Danish, and English source materials, and evaluated by a Norwegian-speaking population. These factors limit generalizability to other linguistic and cultural contexts. Communication norms, empathy expression (Rasool et al., 2025), and information preferences vary substantially across cultures; therefore, performance differences observed here should not be interpreted as universally applicable. External validation in diverse settings is needed.

Questions and responses were sourced from publicly available websites, which may have been included in GPT-4o's training data. However, given the scale and diversity of the training corpus, specific memorization of these items is unlikely. Moreover, the consistent preference for GPT-4o responses suggests that its performance cannot be explained by simple reproduction of memorized content.

Finally, this study evaluated GPT-4o (version 2024-08-06), which, like other large language models, is constrained by its training data and cutoff date and may provide outdated information or reflect corpus biases. As newer models (e.g., GPT-5, Claude Sonnet 4.5, xAI Grok 3) have since been released, the results represent a specific point in time in LLM development and may not fully reflect the capabilities of more advanced systems. While the generalizability of our findings to other models remains uncertain, the dual-perspective evaluation framework and insights regarding educational background effects and quality dimensions are applicable to future model generations. Future studies should include updated evaluations with newer models to assess whether improvements alter the balance between AI- and healthcare generated responses.

### 4.3 Future research

This study gives inspiration for the following aspects to be explored:

- Conduct real-time, interactive studies using LLMs, particularly knowledge-infused models, as support tools for individuals managing chronic conditions such as diabetes, to better assess their performance in dynamic clinical communication contexts.
- Systematically assess the clinical accuracy and safety of LLM-generated responses through expert review by diabetes specialists, identifying potentially harmful content and deviations from current guidelines.
- Conduct longitudinal studies to examine patient knowledge retention, adherence, and clinical outcomes, assessing the real-world educational impact of LLM-assisted communication.
- Expand participant recruitment to include more diverse sociodemographic and health literacy profiles using community outreach and alternative recruitment strategies including community health stations and telephone interviews to improve representativeness and equity.
- Conduct comparative evaluations across multiple LLM systems, generations, and question types to track performance evolution, identify model-specific characteristics, and determine domains where models perform best.
- Investigate the long-term impact of LLM-assisted communication on patient behavior, adherence, and clinical outcomes in longitudinal designs.
- Given the rapid evolution of large language models, systematic comparative evaluation across model generations is essential. Future research should apply the methodological framework established in this study to evaluate GPT-5 and subsequent models, examining whether the patterns we observed (particularly regarding educational background effects and quality dimension correlations), remain consistent across model architectures. Such longitudinal, cross-model comparisons will build cumulative knowledge about how LLM capabilities in healthcare communication evolve over time.

## 5 Conclusion

This study contributes to a growing understanding of how large language models may support health communication and guidance for individuals with chronic conditions such as diabetes. While findings demonstrated statistically significant preferences for GPT-4o responses across key dimensions, the observed effect sizes were small and should be interpreted as modest in practical terms. Results across similar studies remain variable. These factors underscore the need for further research to assess the reliability, safety, and contextual relevance of LLM-generated health information.

Before these models can be responsibly implemented in clinical or patient-facing settings, critical issues must be addressed, including clinical accuracy, ethical considerations, integration with existing care models, and user trust.

It is also important to consider the rapid evolution of LLMs, as their performance may already surpass that of earlier models assessed in this or other studies. Continued evaluation is essential to ensure that their deployment supports, rather than compromises, high-quality and equitable healthcare.

## Data Availability

The datasets presented in this study can be found in online repositories. The names of the repository/repositories and accession number(s) can be found at: https://github.com/alu042/HumanVsRobot.
